# *Ex vivo* transdermal delivery of ^3^H-labelled atovaquone solid drug nanoparticles: a comparison of topical, intradermal injection and microneedle assisted administration†

**DOI:** 10.1039/d3na00454f

**Published:** 2023-10-17

**Authors:** Sam Morris, Mark Long, Alison Savage, Andrew Owen, Steve Rannard, Helen Cauldbeck

**Affiliations:** a Radiomaterials Laboratory, Department of Chemistry, University of Liverpool Crown Street Liverpool L69 7ZD UK helen.cauldbeck@liverpool.ac.uk; b Unilever Research Centre Port Sunlight, Quarry Road East, Bebington Wirral CH63 3JW UK; c Centre of Excellence in Long-acting Therapeutics (CELT), University of Liverpool Liverpool L7 3NY UK; d Department of Pharmacology and Therapeutics, University of Liverpool Liverpool L7 3NY UK; e Materials Innovation Factory, University of Liverpool Oxford Street Liverpool L7 3NY UK

## Abstract

Inherent barrier properties of the skin impose significant challenges to the transdermal delivery of drugs to systemic circulation. Here, the *ex vivo* transdermal permeation and deposition of an anti-malarial prophylactic atovaquone solid drug nanoformulation is radiometrically evaluated following application of a solid microneedle format.

## Abstract 2

*Ex vivo* transdermal permeation and deposition of atovaquone solid drug nanoparticles. Topical administration exhibited limited penetration, intradermal injection delivered a burst release and the microneedle assisted route offered sustained delivery.

## Graphical abstract

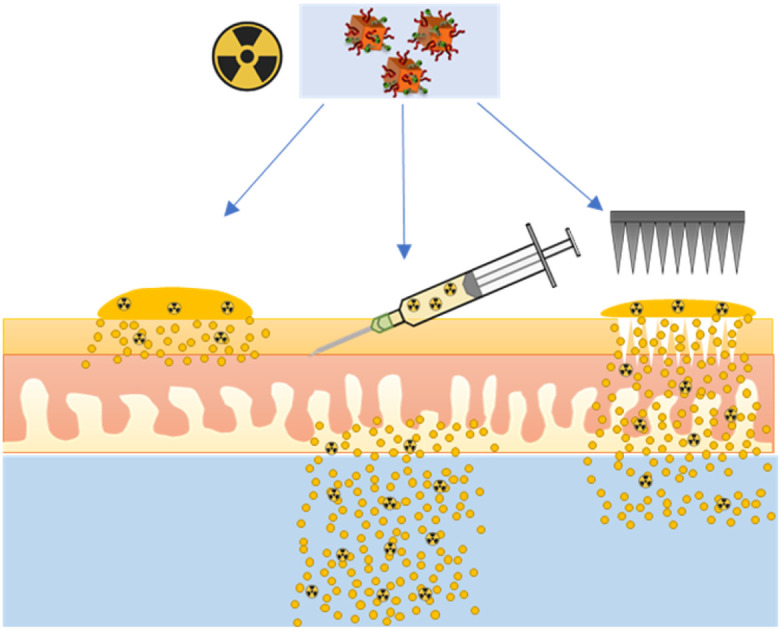

## Introduction

Prevention of malaria by chemoprophylaxis is currently recommended by the World Health Organisation (WHO) to people travelling to malaria endemic areas as well as certain high-risk members of the local population.^1^ Chemoprophylactic regimens recommended by the WHO include Malarone, a combination therapy of atovaquone (ATQ) and proguanil, taken daily as an oral tablet formulation, travellers to endemic regions must begin dosing several days prior to entering an endemic region and continue to medicate 7 days after departure.^1^ Repetitious oral dosing regimens have long been known to manifest issues in patient adherence due to pill fatigue and treatment complacency.^2–4^ Transdermal drug delivery (TDD) has the potential to alleviate some of the burdens of daily oral drug regimens by simplifying the drug administration procedure at the level of the patient,^5–7^ especially if the transdermally dosed active pharmaceutical ingredient (API) exhibits a long-acting, ‘set and forget’ delivery profile.^6,8^ Despite the attractive nature of TDD, the natural barrier function of the skin leads to inherent limitations.^9^ The skin's outer layer – the stratum corneum (SC) – is a stratified layer of dead corneocyte cells held together by a matrix of glycolipid and connective desmosome structures.^10^ Permeation of both hydrophilic and hydrophobic solutes are possible through the SC *via* the intracellular and intercellular pathways respectively,^11,12^ but the efficiency is typically low.^13,14^

ATQ, one of the APIs present in Malarone, is an example of a ‘brick dust’ drug compound, exhibiting exceedingly low aqueous solubility (<0.2 μg mL^−1^)^15^ and low, highly variable oral bioavailability (between 5 and 23% depending on the fasting state of the individual).^15,16^ Topical formulations of lipophilic APIs, such as ATQ carried in polar solvents, are not viable for TDD due to the non-biocompatible and adverse cellular effects of the organic solvents required for their administration.^17^ In the past decade, solid drug nanoparticle (SDN) formulations of lipophilic drugs have displayed a variety of beneficial factors such as aqueous solubility kinetics enhancement,^18^ and increased bioavailability.^19,20^ In 2018, Bakshi *et al.* reported an SDN formulation of ATQ which provided long-acting mono-chemoprophylactic activity *in vivo*;^21^ intramuscular injection of the aqueous based formulation provided favourable curative doses. The work of Bakshi *et al.* demonstrated the effectiveness of enhanced SDN processing and administration compared to the parent drug. Due to the aqueous dispersion of SDNs there is scope for further research into alternative drug delivery regimes for the administration of ATQ, including TDD. TDD of SDNs has the potential to widen the scope of malaria therapeutics and open opportunities for alternative drug delivery systems. An increasing area of interest in TDD is the use of microneedle arrays (MNs), also known as microarray patches, to mechanically breach the SC, epidermis and superficial dermis, therefore bypassing the skin's natural barrier function.^22–25^ A number of different MN technologies exist including hollow,^26–28^ coated,^29,30^ dissolving,^31,32^ hydrogel forming,^33,34^ and solid MNs.^35–38^ Solid MNs consist of sub-millimetre protrusions usually fabricated from metal or silicon.^35^ Solid MNs used in drug delivery act as a permeation enhancer, piercing the SC in preparation for a transdermal dose of drug which is introduced directly on top of the pores created by the MN. This type of MN-assisted TDD has been shown to increase the transdermal flux of many therapeutic molecules.^39,40^

**Fig. 1:**
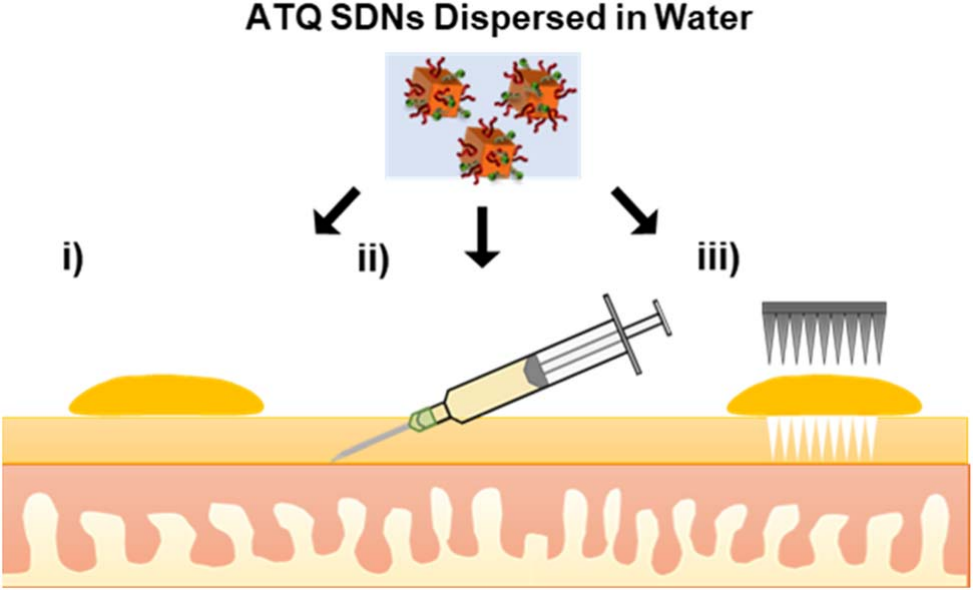
Alternative transdermal drug delivery administration routes of an aqueous SDN dispersion of atovaquone (i) topical, (ii) intradermal injection and (iii) solid microneedle assisted dosing. (Objects not to scale).

In this work, tritium-labelled (^3^H) ATQ SDNs have been prepared for the radiometric evaluation of the TDD of SDNs. Topical, intradermal injection and solid MN assisted administration routes of ^3^H-labelled aqueous SDN dispersions have been dosed to an *ex vivo* porcine skin model, Fig. 1. Here we demonstrate the penetration and deposition of ^3^H-labelled ATQ SDNs within an *ex vivo* skin model using a Franz diffusion cell approach. Our results show how the various administration routes direct drug diffusion and distribution profiles and provide a greater understanding of the TDD of such nanoformulations. The suitability of solid MNs for the long-acting TDD of ATQ SDNs and their potential to achieve malaria prophylaxis will be discussed.

## Results and discussion

To monitor the TDD of ATQ, a radiolabelled water-dispersible nanoformulation of the poorly water-soluble drug was required. SDNs of ^3^H-labelled ATQ were prepared at an 80 wt% ATQ content *via* emulsion-templated freeze drying, as previously reported.^21^ Nanoparticles were dispersed to give a clear yellow aqueous nanodispersion and analysed by dynamic light scattering (Malvern Zetasizer Nano ZS) using automatic measurement optimisation; the nanoparticles showed a *Z*-average hydrodynamic diameter of 355 nm, incorporation of ^3^H ATQ did not affect the SDN formulation (ESI Fig. S1–S3 and Table S1†). Radiolabelling of ATQ SDNs enables facile and accurate quantification of drug permeation and deposition without the need for complex extraction methods, and removes the need for conjugated fluorescent markers, which would alter the chemistry of the analyte and consequently influence drug permeation by modifying interactions between API and dermal tissue. An aqueous ^3^H-labelled ATQ SDN dispersion (100 μL, 1 mg mL^−1^, 0.947 MBq mg^−1^) was administered to porcine skin *via* (i) topical, (ii) intradermal injection performed at <15° angle, to an injection depth of 60 μm, to ensure the dermal layer was targeted (25G hypodermic needle), and (iii) solid MN (metal, 32G, 12 needle array, inserted 0.5 mm, Fig. S4†) assisted administration routes (56.5 μg cm^−2^, 53.5 kBq cm^2^, Fig. 1).

*Ex vivo* porcine skin, a by-product of the meat industry, was obtained from a local abattoir (Morphets Abattoir, Cronton, Merseyside) and used immediately. Studies have shown that *in lieu* of freshly excised human skin, fresh porcine skin provides a reliable model for the estimation of the *in vivo* dermal absorption and penetration of substances within human skin due to its physiological similarity and comparative ease of availability.^41,42^

**Fig. 2:**
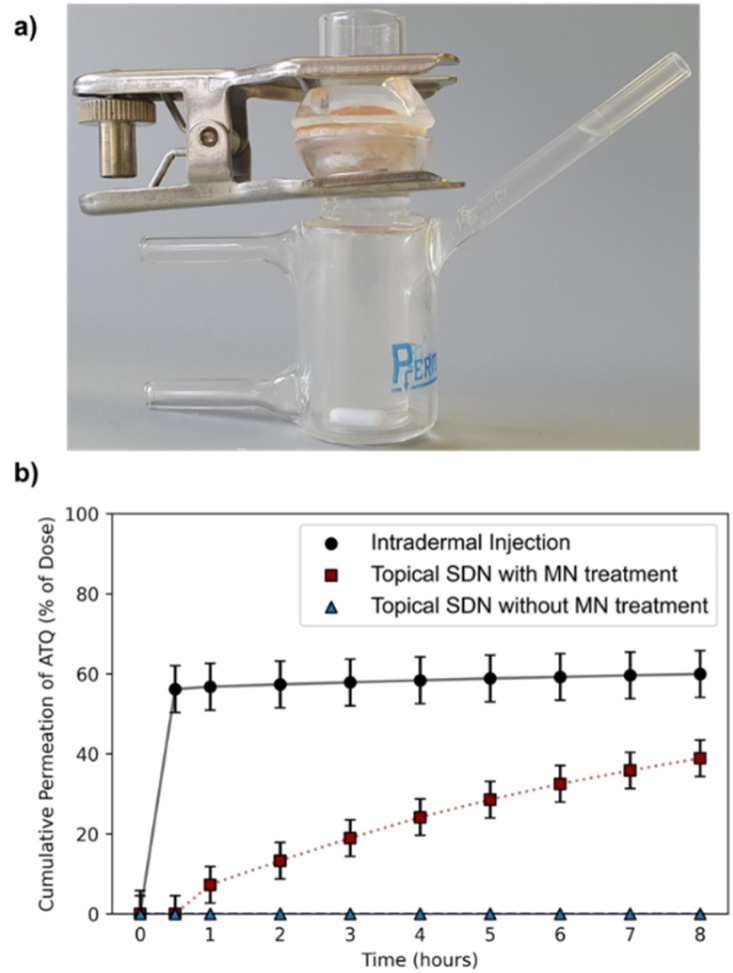
(a) Image of a Franz cell model containing a porcine skin membrane. (b) Cumulative amount of ATQ permeation through *ex vivo* porcine skin into a PBS reservoir following topical (blue triangle), intradermal injection (black circle) and solid MN assisted (red square) administration routes of ^3^H-labelled ATQ SDNs. Results are shown as a percentage of the initial applied dose.

The porcine skin samples were prepared using a Dermatome 75 mm (Nouvag, Goldach, Switzerland) to a thickness of 4.5 mm and cut into discs using a cylindrical corer tool and the discs were affixed to the Franz cell apparatus (PermeGear, 15 mm) to create a barrier between the donor and receptor chambers, Fig. 2a. The SC was exposed in the donor chamber under occlusive conditions, whilst the receptor chamber contained a reservoir of phosphate buffered saline (PBS) to allow a measurement of ATQ permeation through the skin. Sampling was performed over an 8 h time period; aliquots of 1 mL were taken from the reservoir solution and replenished with fresh PBS. The amount of ATQ which had permeated through the skin was determined by direct sampling of the reservoir solution; due to the radioactive specific activity of the sample the amount of ATQ can be determined even at extremely low concentrations. The amount of ATQ released into the PBS reservoir was quantified *via* liquid scintillation counting using a PerkinElmer 3100TR and tritium cocktail (ProSafe+, Meridian Biotechnologies Ltd, 10 mL), and radioactivity measured.

Permeation of ^3^H-labelled ATQ SDNs through the *ex vivo* skin varied greatly depending on the administration route, Fig. 2b. The permeation profiles obtained clearly demonstrate that topical administration of the aqueous dispersion of ATQ SDNs attained a significantly lower level of diffusion through the skin than that achieved using alternative administration routes.

For example, <0.1% of the ATQ within the topically applied dose had permeated to the reservoir after 1 hour, whereas 57% of ATQ SDNs administered *via* intradermal injection was detected within the reservoir over the same time period. Interestingly, when the skin was pre-treated with MNs, prior to the application of a topical dose, there was a clear transdermal flux of the ATQ nanoformulation compared to a standard topical dose. The release profile showed a sustained release of 39% of the ATQ payload over the 8 hours to the reservoir. The steady-state transdermal flux of ATQ for each administration route was calculated based on Fick's law of diffusion *via* the construction of graphical plots of cumulative mass released per area of skin over time, ESI eqn (S1.1) and Fig. S5,† in accordance with previously reported methods.^40,43–45^ The transdermal flux of ATQ was calculated to be 9 × 10^−4^, 0.2661 and 2.573 μg cm^−2^ h^−1^ for topical, intradermal and MN assisted transdermal dosing, respectively. These values clearly demonstrate MN assisted TDD is superior to topical and intradermal injection administration for a long-acting therapy and allows the sustained release of higher doses for a longer time period. There was however an initial 30-minute delay after MN assisted topical dosing before ATQ was detected in the reservoir. This retardation in drug permeation following MN assisted administration is likely due to the MN insertion depth (600 μm).

Overall, this data exemplifies the excellent barrier properties of the skin to xenobiotics and the ability of MNs and intradermal injections to overcome this, however MNs also eliminate the burst release profile following intradermal injection. The dose kinetics of topical dose administration following solid MN insertion are therefore tailored towards a longer acting, more sustained TDD therapy.

**Fig. 3:**
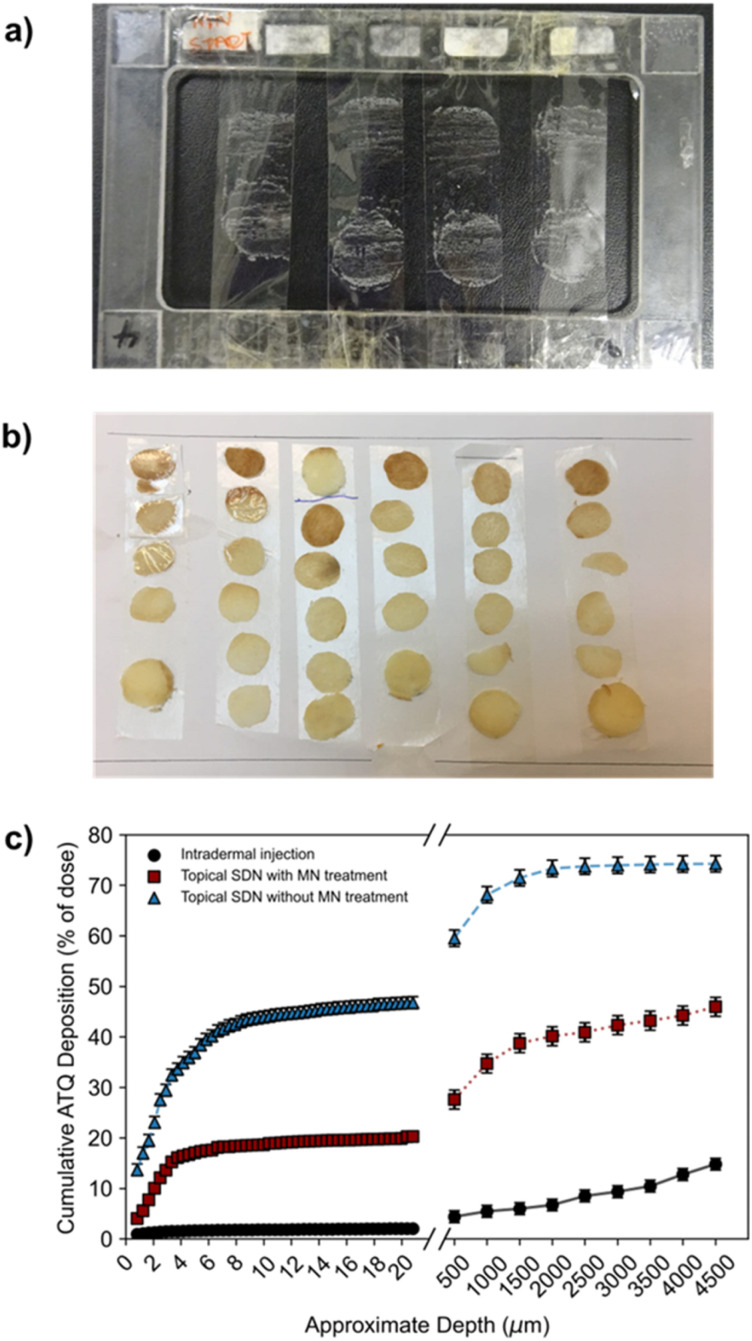
(a) Tape strips collected of *ex vivo* porcine stratum corneum. (b) Vibratome sections of porcine skin of 500 μm depth. (c) The deposition profile of ^3^H-labelled ATQ SDNs. The deposition quantification of ^3^H-labelled ATQ SDNs following topical (blue triangle), intradermal injection (black circle) and solid MN assisted (red square) administration routes of ^3^H-labelled ATQ SDNs. Results are shown as a percentage of the initial applied dose.

To determine a spatial mass balance of the remaining dose, deposition profiles of ^3^H-labelled ATQ within the porcine skin discs were studied. This technique depended on the use of ^3^H-labelled ATQ and required sectioning of the skin *via* the use of tape stripping and vibratome techniques. Tape stripping is a simple and efficient method for the removal of cell layers of the SC which allows the penetration depths of APIs to be determined. To ensure the uniform removal of the SC in both lateral and vertical directions a stamp was applied with equal force for the same time period for each tape strip. A total of 50 tape strips were collected per sample to remove 75–95% of the SC, Fig. 3a.^46,47^ The average thickness of a porcine SC is 26 μm,^48^ therefore, tape stripping led to the removal of *ca.* 20 μm of the porcine SC. The remaining dermal tissue was sectioned using a vibratome at sequential depths of 500 μm, Fig. 3b. Tape strips and skin sections were dried under ambient conditions for 24 h before oxidation at 1300 °C to allow the collection of the resulting ^3^H_2_O, a combustion product which directly correlates to, and quantifies, the presence of ^3^H-labelled ATQ in the samples. ^3^H_2_O was quantified *via* LSC to determine ATQ content as a function of layer depth, Fig. 3c.

The deposition profiles further exemplified the barrier function of the SC. The unassisted topical dose of ATQ SDNs were shown to concentrate within the first 20 μm of the SC, with 42% of the initial dose within the top 8 μm, and a depleting concentration of ATQ SDNs detected at increased skin depth.

This deposition profile, in combination with the permeation concentration recovered of the topically applied dose (0.1%), showed the transdermal topical administration of ATQ SDNs is not viable; the majority of the topical dose fails to permeate through the SC. Conversely, intradermal injection administration showed a relatively low amount of ATQ within the skin section, especially within the first 20 μm of the SC, 2%. This was expected, as the purpose of an intradermal injection is to bypass the SC entirely, delivering the dose directly into the dermis. Any ATQ dosed remaining in the SC (2%) is likely due to three-dimensional diffusion of the injection bolus that includes diffusion towards the SC, as well as deposition of trace amounts of drug from the needle tip during injection. MN administration of ATQ SDNs displayed a vastly different deposition profile. Here, 20% of the dose was located within the SC, with an additional 27% distributed throughout the remaining skin section. The high concentration of dose within the SC is likely due to the limited volume of the pores created from the MN insertion and the efficiency of the pores being filled with SDN dispersion, as well as wastage of a portion of the dose once the carrier aqueous vehicle evaporates. This dosing technique, however, did show a much more even distribution of ATQ throughout the skin showing that the pre-treatment of skin with solid MNs followed by a topical dose improved the deposition and permeation profiles significantly when compared with a traditional topical dose. Importantly, long-acting release kinetics are observed for MN assisted topical dosing whilst also displaying permeation concentrations comparable to an intradermal injection.

The deposition profiles of both the topical and intradermal administration routes represented two extremes of ATQ permeation through the skin. Topical TDD showed ATQ was sequestered on top of and within the SC, resulting in permeation of an extremely limited dose to the reservoir over the course of 8 hours. In contrast, intradermal injection of the nanoformulation led to a burst release of drug through the skin, releasing 56% of the dose within 30 minutes of administration. The deposition profile of the MN-assisted dose occupies the middle of these extremes, displaying a gradual permeation of ATQ through the skin and maintaining a more even distribution of drug throughout the skin. Despite this improved permeation profile of the MN-assisted dose, there are still fundamental limitations associated with the application of subsequent topical dosing. Indeed, in both topical dosing routes in this study there was a significant recovery of drug within the first 2 layers of SC collected *via* tape stripping. For the MN assisted dose of ATQ SDNs 4% of the dose remained on the skin surface, and 7.3% for the topical dose, ESI Fig. S6.† It is possible the use of a larger MN array could reduce this issue for MN assisted transdermal dosing.

## Conclusions

**Fig. 4:**
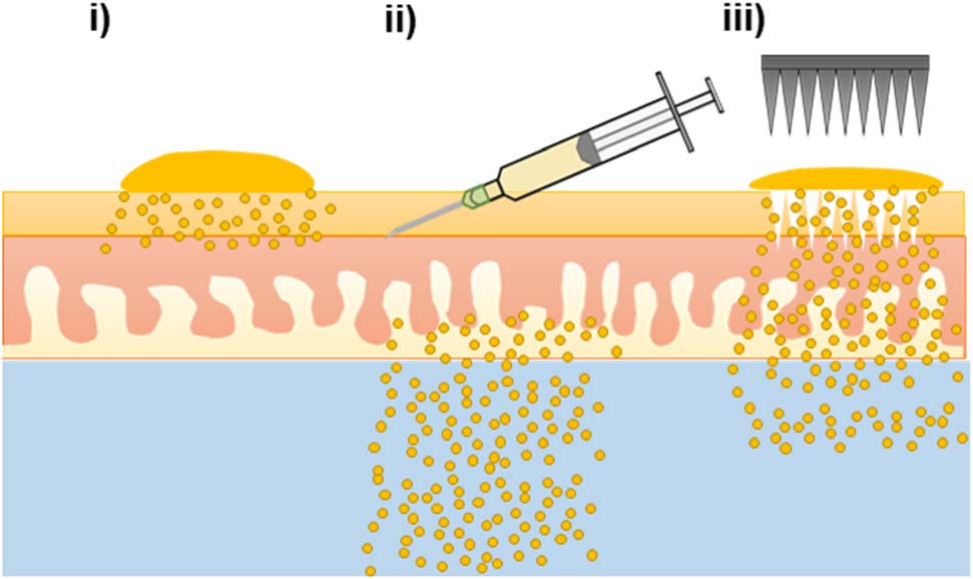
Schematic representation of atovaquone permeation and deposition profiles through *ex vivo* porcine skin following transdermal drug delivery administration routes of an aqueous SDN dispersion of atovaquone (i) topical, (ii) intradermal injection and (iii) solid microneedle assisted followed by topical dose. (Objects not to scale).

The nanoformulation of ATQ, a hydrophobic, antimalarial chemoprophylactic, into SDNs provided an aqueous dispersion of the drug which could be used for TDD. Without the nanoformulation of ATQ, TDD would not be possible as polar, non-biocompatible solvents would be needed to solubilise the drug. Here, the first example of TDD of SDNs dosed *via* topical, intradermal injection and MN assisted application were assessed *ex vivo* by radiometric evaluation. The ability to alter drug delivery profiles of transdermally applied SDNs by using varied administration routes, Fig. 4, may have considerable value to posology of ATQ *in vivo*. Whilst intradermal injection has been shown to provide a burst release of ATQ, a less invasive MN assisted route, which offers a sustained delivery, may provide the opportunity for less frequent dosing.

In addition to the variation in TDD created by varying the drug administration route, the longevity of release from a nanoformulation of ATQ SDN compared the parent drug would allow the time between antimalarial drug dosing to be prolonged. The drug release characteristics of the MN-assisted transdermal dose closely followed a longer-acting release profile, demonstrating the applicability of SDN formulation and MN technologies in the tailoring of drug delivery towards a more controllable, sustained delivery regime. The permeation enhancement effect of MN pre-treatment is still limited however, by the diffusion of the drug from the skin surface into the MN pores.

## Conflicts of interest

AS, AO and SR have a filed patent for the ATQ SDN technology used.

## Supplementary Material

NA-005-D3NA00454F-s001
