# Supplementary material for: Ex vivo transdermal delivery of 3H-labelled atovaquone solid drug nanoparticles: a comparison of topical, intradermal injection and microneedle assisted administration
Source: Nanoscale Adv. 2023 Oct 17;5(23):6400–4. doi: 10.1039/d3na00454f (PMC10662085; doi:10.1039/d3na00454f)
Supplement: NA-005-D3NA00454F-s001 [file NA-005-D3NA00454F-s001.pdf]

Electronic Supplementary Material (ESI) for Nanoscale Advances.
This journal is © The Royal Society of Chemistry 2023

# Supplementary Information

# Ex vivo transdermal delivery of 3H-labelled atovaquone solid drug nanoparticles: a comparison of topical, intradermal injection and microneedle assisted administration

Sam Morris,[a] Mark Long,[b] Alison Savage,[c] Andrew Owen,[c,d] Steve Rannard,[c,e] Helen Cauldbeck[c,a*]

## Experimental

### Formulation of atovaquone solid drug nanoparticles

ATQ SDNs with a drug-loading of 80 wt% were formulated following a previously established emulsion-templated freeze-drying based approach.[1] Stock solutions of 80 mg $mL^{-1}$ atovaquone in chloroform, 22.5 mg $mL^{-1}$ poly (vinylpyrrolidone) (PVP K30; average mol. wt. 40 000 g $mol^{-1}$) in water, and 22.5 mg $mL^{-1}$ poly (ethylene oxide)$_{20}$ sorbitan monooleate (Tween 80) in water were prepared. Oil-in-water mixtures were prepared in the proportion of 100 mL atovaquone, 63.7 mL PVP K30, 31.5 mL Tween 80, and 304.5 mL distilled water to prepare SDNs with a solid mass ratio of 80 % atovaquone, 13 % PVP K30 and 7 % Tween 80 and an oil:water ratio of 1:4. The mixtures were emulsified using a Covaris S2x for 30 s with a duty cycle of 20, intensity of 10 and 500 cycles/burst in frequency sweeping mode before immediate freezing in liquid nitrogen. Samples were lyophilised using a Virtus Benchtop K freeze-drier for 42 h to leave dried porous monoliths which were sealed in individual vials prior to analysis/use. To prepare $^{3}$H-labelled ATQ SDNs, $^{3}$H ATQ was added to the chloroform stock solution prior to formulation and the SDNs were formulated at half-scale (50 mL atovaquone, 31.85 mL PVP K30, 15.75 mL Tween 80, and 152.4 mL distilled water).

### Analysis of atovaquone solid drug nanoparticles

Dynamic light scattering (DLS) analysis of the ATQ SDNs dispersed in water to an ATQ concentration of 0.5 mg $mL^{-1}$ was performed (Malvern Zetasizer Nano ZS) using automatic measurement optimisation; immediately after dispersion and vigorous sample agitation using a vortex mixer. For $^{3}$H-labelled SDNs, the target formulation was prepared at half-scale (i.e. for a solid mass of 4 mg ATQ) and submitted for DLS analysis at 0.5 mg $mL^{-1}$ to assess reproducibility at this scale. DLS quality criteria were as follows: complete aqueous dispersion at 0.5 mg $mL^{-1}$; Z-average diameter < 500 nm; polydispersity index < 0.4; Z-average diameter < 5% SD between three measurements. DLS analysis of 80 wt% ATQ SDNs at both full- and half-scale revealed the congruence of data at both scales and

indicated their suitability for *ex vivo* studies. Z-average diameters, PDI and SD% values are provided in ESI Figure S1.

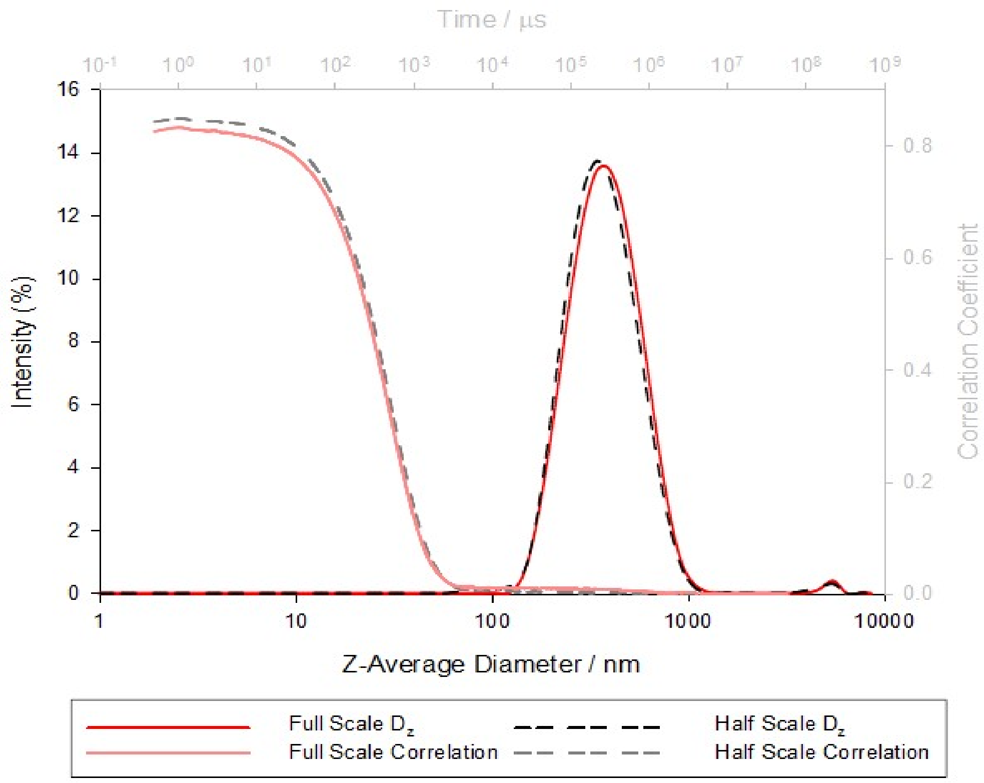


Figure S1. ATQ SDN size distribution obtained by DLS analysis with corresponding correlogram.

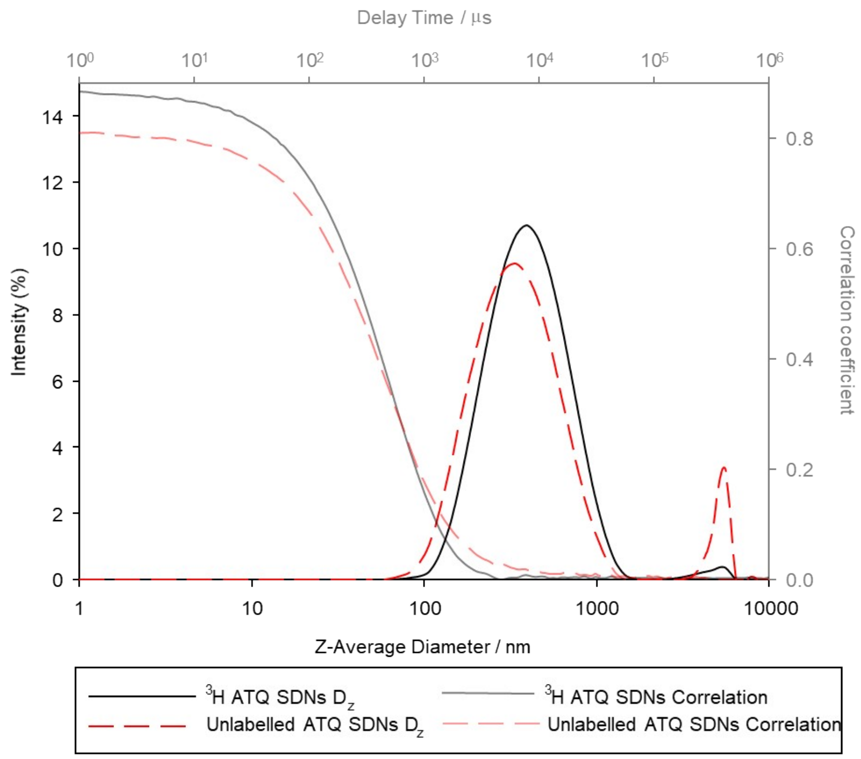

Figure S2. $^{3}H$ ATQ SDN size distribution obtained by DLS analysis with corresponding correlogram.

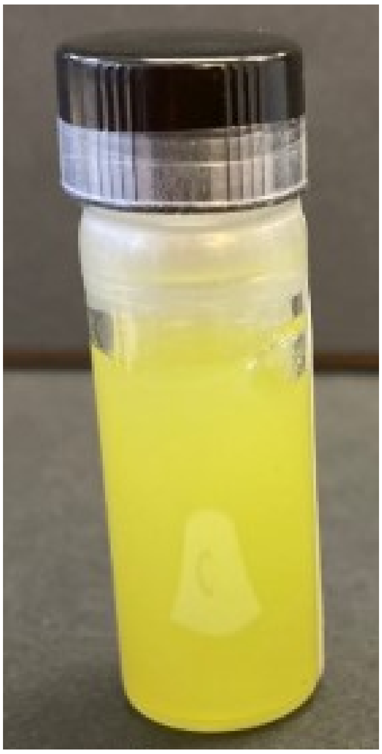

Figure S3. Visual observation of ATQ SDN aqueous nanodispersion.

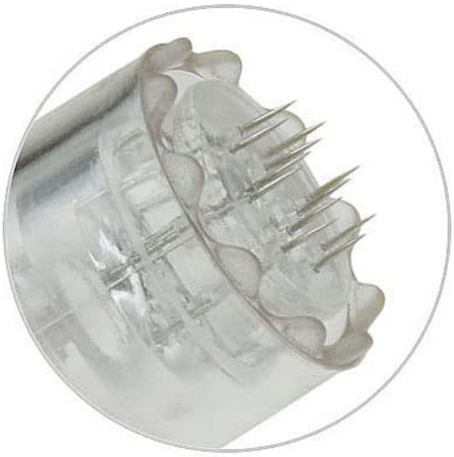

Figure S4. Image of TBPHP solid microneedles used for pre-treatment of *ex vivo* skin.

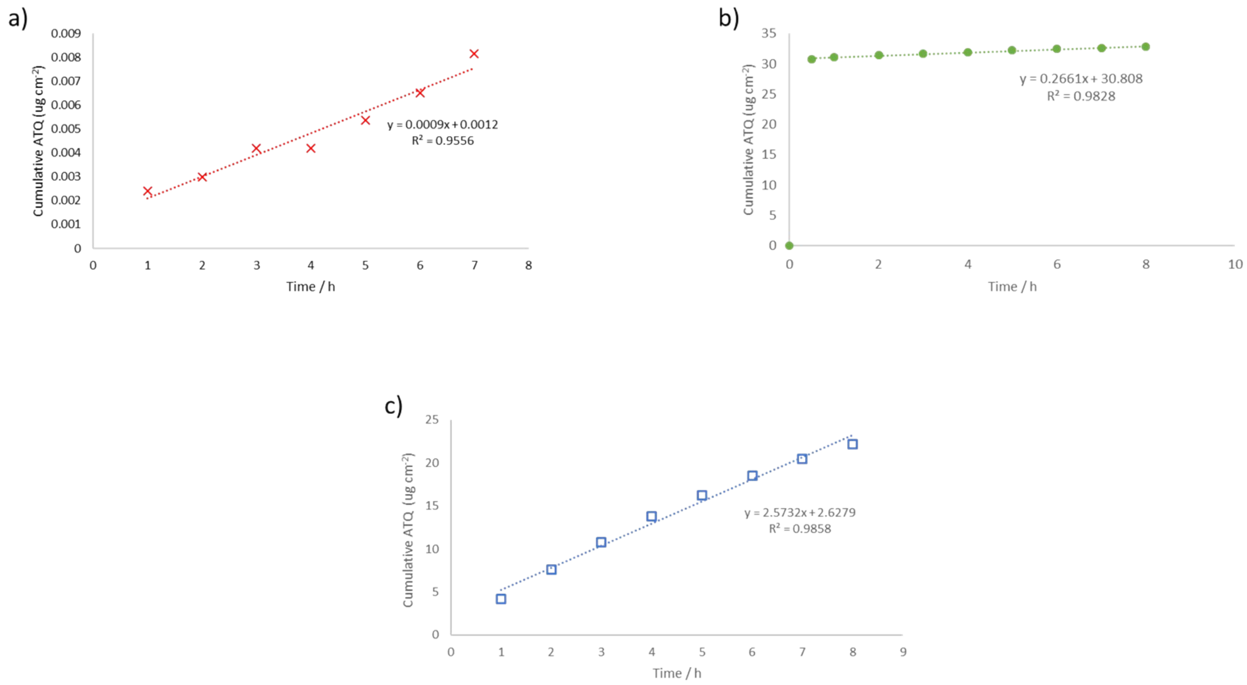


Figure S5. Graphical plots of cumulative mass released per area of skin over time to determine the steady-state transdermal flux of atovaquone *via* a) topical, b) intradermal injection and c) MN-assisted administration. Steady-state transdermal flux is equal to the gradient.

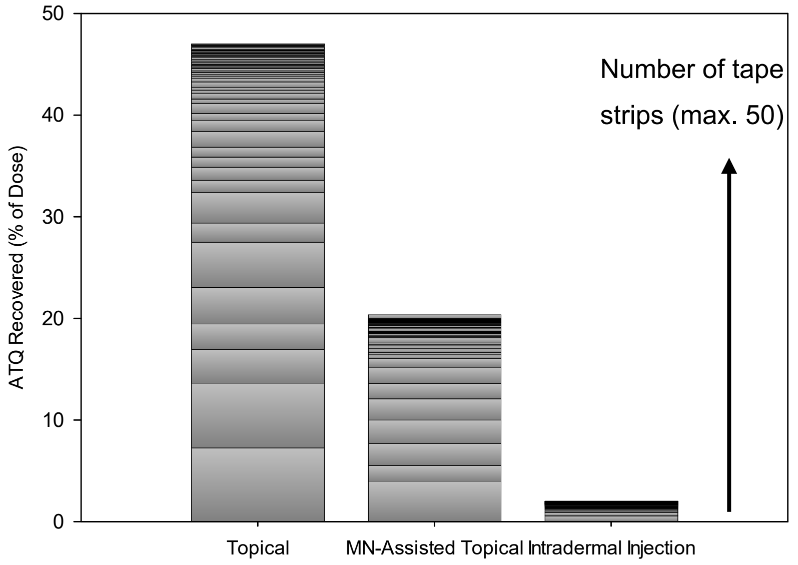


Figure S6. Graphical representation of atovaquone dose recovered with subsequent tape strips collected and analysed.

Table S1. DLS analysis of aqueous nanoparticle dispersions produced of ATQ.

| ATQ | Dz (nm) | PDI | S.D (%) |
|---|---|---|---|
| Unlabelled | 360± 4 | 0.45 | 4.05 |
| $^3$H | 355± 15 | 0.21 | 1.14 |

$$\frac{1}{A}\left(\frac{dM}{dt}\right) = J_s$$

Equation S1

$J_s$ = Flux at steady state

$A$ = Area of the skin (1.77 $cm^2$)

$M$ = Cumulative amount of drug permeating through the skin

$t$ = Time
